# Epidemiology of Bluetongue Virus and Epizootic Hemorrhagic Disease Virus in Beef Cattle on a Ranch in South-Central Florida

**DOI:** 10.1089/vbz.2018.2406

**Published:** 2019-09-26

**Authors:** Mary M. Merrill, Raoul K. Boughton, Laurent O. Lollis, Katherine A. Sayler, Samantha M. Wisely

**Affiliations:** ^1^ Department of Environmental and Global Health, College of Public Health and Health Professions, University of Florida, Gainesville, Florida.; ^2^ Range Cattle Research and Education Center, University of Florida, Ona, Florida.; ^3^ Buck Island Ranch, MacArthur Agro-Ecology Research Center, Archbold Biological Station, Lake Placid, Florida.; ^4^ Department of Wildlife Ecology and Conservation, Institute of Food and Agricultural Sciences, University of Florida, Gainesville, Florida.

**Keywords:** bluetongue virus, cattle, epidemiology, epizootic hemorrhagic disease virus

## Abstract

Bluetongue virus (BTV) and epizootic hemorrhagic disease virus (EHDV) infect a variety of wild and domestic ruminant hosts in the United States, with outcomes ranging from subclinical infection to clinical disease resulting in mortality. Because cattle have been suggested as a temporary reservoir for both BTV and EHDV, ongoing national surveillance for these viruses may benefit from inclusion of domestic cattle as a supplement to current programs, such as surveillance of wild white-tailed deer. To better understand the prevalence of BTV and EHDV in cattle, we surveyed for viral RNA (vRNA) in the blood of 1,604 beef cattle on a south-central Florida cattle ranch over 3 years. While overall prevalence of vRNA in blood was low (<2% for either virus), the occurrence of vRNA was much higher in young animals: in 2016, 24% of animals 2 years old were positive by PCR for either BTV or EHDV. Our results suggest that cattle are a likely temporary reservoir for these viruses in Florida, and could provide additional information on the spatial distribution, viral diversity, and timing of emergence of these viruses, particularly if surveillance was restricted to cattle ≤2 years of age.

## Introduction

Bluetongue virus (BTV) and epizootic hemorrhagic disease virus (EHDV), both members of the genus *Orbivirus* in the family *Reoviridae*, are responsible for disease outbreaks among wild ruminants, particularly white-tailed deer (*Odocoileus virginianus*), in the United States (Savini et al. 2011, Maclachlan et al. 2015a, Ruder et al. 2015). A wide range of domestic and wild ruminants throughout much of the world are susceptible to infection with these viruses, with outcomes ranging from subclinical infection to severe morbidity and mortality (Savini et al. 2011, Maclachlan et al. 2015a). Although BTV infection does not typically cause disease in cattle, disease can result when naïve populations are exposed (Maclachlan et al. 2015a). Cattle that do show signs usually present with lethargy and inappetence, although reproductive disorders have been documented such as those in the recent BTV-8 outbreaks in Europe (Dal Pozzo et al. 2009). Like BTV, clinical signs of EHDV infection are rarely observed in cattle, although certain serotypes or strains of the virus are capable of causing disease and may result in production losses (Howerth et al. 2001, Kedmi et al. 2010, Savini et al. 2011). For example, a study of dairy cattle during an EHDV outbreak in Israel showed that increased EHDV seroprevalence was associated with decreased milk production and increased mortality (Kedmi et al. 2010).

In the United States, white-tailed deer have been used for decades as sentinels for these viruses. White-tailed deer are excellent sentinels because they often develop disease, are present throughout much of the United States, are accessible for study through programs such as hunter check stations, and are already incorporated into existing wildlife disease surveillance programs at Southeastern Cooperative Wildlife Disease Study and the United States Department of Agriculture's National Veterinary Services Laboratories (Ruder et al. 2015). However, additional methods of surveillance such as the use of domestic cattle and captive (farmed) white-tailed deer have been suggested to supplement ongoing programs (Ruder et al. 2015). Cattle are a potential temporary reservoir host of BTV and EHDV in the United States (Maclachlan et al. 2015b), and may supplement existing surveillance programs to improve understanding of virus distribution (Ruder et al. 2015) and emergence of imported serotypes of these viruses.

In south-central Florida, the high regional seroprevalence of BTV in cattle and the high concentration of beef cattle herds provide a unique opportunity for BTV and EHDV investigation in domestic livestock (Gibbs and Greiner 1985). More than a dozen BTV serotypes and three serotypes of EHDV have been detected in Florida (Subramaniam et al. 2017, Ahasan et al. 2018, Schirtzinger et al. 2018), and the state hosts multiple *Culicoides* species that are confirmed or suspected vectors of BTV or EHDV (Mellor et al. 2000, Ruder et al. 2015). Florida is one of the top 15 beef cattle producing states in the United States, with combined breeding herd and calf values exceeding U.S. $1 billion (Florida Department of Agriculture and Consumer Services 2012). Not much is known about the potential production effects that BTV and EHDV serotypes already present in Florida may have on beef cattle. A greater understanding of the current distribution, prevalence, and transmission patterns of BTV and EHDV in Florida cattle is needed to inform both wildlife and domestic animal health. We sampled beef cattle over 3 years on a ranch in south-central Florida to determine BTV and EHDV infection prevalence and associations of infection with sampling date, age, and pregnancy status.

## Materials and Methods

### Study site

The MacArthur Agro-ecology Research Center (MAERC), a division of Archbold Biological Station, is located at Buck Island Ranch (BIR) in Lake Placid, Florida (Swain et al. 2013). BIR is a full-scale commercial beef cow-calf operation and one of Florida's top 20 beef cattle producers, with >3,000 head of cattle on 4,249 ha (Swain et al. 2013). BIR hosts many native wildlife species such as white-tailed deer (*O. virginianus*), wild turkey (*Meleagris gallopavo*), and Northern bobwhite (*Colinus virginianus*), as well as some invasive exotic species such as wild pigs (*Sus scrofa*).

### Sample collection

Cattle were mechanically restrained in cattle chutes by ranch staff for routine handling purposes, and as such sampling was opportunistic. Sampling occurred in June 2015; May, August, and November 2016; and February, March, and August 2017. In North America, BTV and EHDV typically have seasonal transmission patterns, beginning in late-summer and lasting through autumn, but transmission in the tropics may last most of the year (Maclachlan et al. 2015a, Ruder et al. 2015). Serological data from the 1980s detected BTV seroconversion of cattle in Florida from early summer to mid-winter, varying by study site (Gibbs et al. 1983, Gibbs and Greiner 1985). Twelve milliliters of blood were drawn from the jugular or tail vein of cattle, and 1–2 mL were collected into EDTA-coated tubes. Blood samples were stored at 4°C until transferred to −80°C for long-term storage.

For each animal, age and pregnancy status (of breeding age females exposed to bulls, artificial insemination, or embryo transfer) were recorded. Animal age at the time of sample collection was calculated as the year of the sampling date minus the birth year of each animal. In this study, we used the term “calf” to refer to an animal <1 year of age. Pregnancy status was determined by hand palpation or sonogram by experienced ranch or veterinary staff. Pregnancy status was recorded and used in statistical analysis as disease caused by BTV and EHDV has in rare cases been associated with abortion, and EHDV has been suggested to affect fertility, in cattle (Savini et al. 2011, Maclachlan et al. 2015a, 2015b). Sample collection was approved by University of Florida Institutional Animal Care and Use Committee (Protocol #201508865).

### RNA extraction and multiplex real-time reverse transcription-PCR

High-throughput isolation of total RNA from whole cattle blood was performed using the MagMAX mirVana Total RNA Isolation Kit (Applied Biosystems, Foster City, CA) with the KingFisher Flex Purification System (Thermo Fisher Scientific, Waltham, MA). We included a commercially available nucleic acid internal control (VetMAX Xeno Internal Positive Control RNA; Applied Biosystems) in each RNA isolation according to manufacturer's instructions. Nucleic acids were eluted in 50 μL of elution buffer and immediately frozen at −80°C. A previously described multiplexed real-time reverse transcription-PCR (RT-PCR) assay was used to detect BTV and EHDV RNA (Wernike et al. 2015). The NS3 gene of BTV was targeted using forward primer BTV-NS3-183F and reverse primer BTV-NS3-288R, and the NS1 gene of EHDV was targeted using forward primer EHD NS1 5F and reverse primer EHD NS1 80R (Wernike et al. 2015). Fluorescent probes BTV-NS3-242-FAM and EHD NS1 27TEX were used for detection of BTV RNA and EHDV RNA, respectively (Wernike et al. 2015).

We performed RT-PCR assays on the Applied Biosystems 7500 FAST machine (Applied Biosystems) using the VetMAX-Plus Multiplex One Step RT-PCR Kit with 5 μL of template RNA, 1 μL of BTV-NS3-183F at 10 μM, 1 μL of BTV-NS3-288R at 10 μM, 0.2 μL of BTV-NS3-242-FAM at 10 μM, 0.75 μL of EHD NS1 5F at 20 μM, 0.75 μL of EHD NS1 80R at 20 μM, and 0.125 μL of EHD NS1 27TEX at 20 μM to a final reaction volume of 25 μL. The VetMAX Xeno Internal Positive Control VIC Assay, a proprietary primer–probe mix, was included in RT-PCRs to detect internal positive control RNA, according to manufacturer's instructions. Cycling conditions were as follows: 48°C for 10 min, followed by 95°C for 10 min, then 40 cycles of 95°C for 15 s, 57°C for 45 s, and 68°C for 45 s. Positive controls consisted of RNA purified from culture isolates from BTV- or EHDV-infected white-tailed deer. Both BTV- and EHDV-positive controls were included in each RT-PCR assay. Molecular-grade water was included as a negative control in all RNA isolations and RT-PCR assays. Isolation of RNA, preparation of RT-PCR reagents, and execution of RT-PCR assays were performed in separate rooms to prevent contamination. Samples with BTV or EHDV *C_t_* values <38 were considered positive without additional confirmation. Samples with BTV or EHDV *C_t_* values ≥38 were tested in triplicate and considered positive if amplification of the target was detected in at least two of three replicates.

We considered a RT-PCR positive sample indicative of recent (within the past 5–6 months) infection with BTV or EHDV, not necessarily an active infection in the animal. BTV RNA has been shown to be detected in cattle blood by reverse-transcriptase PCR up to 167 days postinfection, months after infective virus was recoverable through virus isolation techniques (Katz et al. 1994, MacLachlan et al. 1994, Bonneau et al. 2002), due to viral sequestration in the cell membranes of red blood cells (Brewer and MacLachlan 1992, MacLachlan et al. 1994). Although viral RNA (vRNA) may remain in ruminant blood for several months, cattle eventually clear the virus and are not truly persistently infected (Katz et al. 1994, Schwartz-Cornil et al. 2008, Maclachlan et al. 2015a).

### Statistical analysis

We tested for an association of BTV or EHDV detection with age and pregnancy status using Pearson's chi-squared test for independence, and when appropriate, applied Yates' continuity correction. We further used logistic regression to quantify the relationships of variables found to be significantly associated with BTV or EHDV detection. Prevalence of infection with BTV (or EHDV) was defined as the proportion of cattle from which BTV (or EHDV) was detected among all cattle sampled during the specified time period (daily, monthly, yearly, or throughout the entire study). Confidence intervals (CIs) for prevalence were calculated using the nonparametric bootstrap with 2,000 replicates (Davison and Hinkley 1997, Canty and Ripley 2016). Data were analyzed in R version 3.3.2 (R Core Team 2016).

## Results

From 2015 to 2017, we detected BTV in 16 animals (1% prevalence, 95% CI: 0.6–1.5) and EHDV in 15 animals (0.9% prevalence, 95% CI: 0.5–1.4) from a total 1,604 animals sampled (Table 1). We detected no BTV or EHDV coinfections in this study. As we detected no EHDV in 2015 or 2017 and detected only one BTV-positive sample each of these years, we excluded 2015 and 2017 from further statistical analyses for lack of data.

**Table 1.** Prevalence of Bluetongue Virus and Epizootic Hemorrhagic Disease Virus Detection Among Cattle Sampled from 2015 to 2017 in South-Central Florida

	*Total sampled*	*No. BTV positive*	*BTV prevalence (%)*	*BTV prevalence 95% CI*	*No. EHDV positive*	*EHDV prevalence (%)*	*EHDV prevalence 95% CI*
2015	287	1	0.3	0.0–1.1	0	0.0	0.0–0.0
June 10	148	1	0.7	0.0–2.2	0	0.0	0.0–0.0
June 11	139	0	0.0	0.0–0.0	0	0.0	0.0–0.0
2016	792	14	1.8	0.9–2.8	15	1.9	0.9–2.9
May	238	9	3.8	1.5–6.5	0	0.0	0.0–0.0
May 17	36	4	11.1	2.3–22.7	0	0.0	0.0–0.0
May 23	202	5	2.5	0.5–4.8	0	0.0	0.0–0.0
August	242	5	2.1	0.4–4.0	11	4.5	2.0–7.3
August 15	117	5	4.3	0.9–8.2	7	6.0	2.0–10.4
August 30	125	0	0.0	0.0–0.0	4	3.2	0.7–6.5
November	312	0	0.0	0.0–0.0	4	1.3	0.3–2.7
November 8	123	0	0.0	0.0–0.0	2	1.6	0.0–4.2
November 9	41	0	0.0	0.0–0.0	2	4.9	0.0–12.5
November 10	148	0	0.0	0.0–0.0	0	0.0	0.0–0.0
2017	25	1	0.2	0.0–0.6	0	0.0	0.0–0.0
February 9	18	0	0.0	0.0–0.0	0	0.0	0.0–0.0
March 21	281	0	0.0	0.0–0.0	0	0.0	0.0–0.0
August	226	1	0.4	0.0–1.4	0	0.0	0.0–0.0
August 28	96	0	0.0	0.0–0.0	0	0.0	0.0–0.0
August 29	130	1	0.8	0.0–2.5	0	0.0	0.0–0.0
Overall	1,604	16	1.0	0.6–1.5	15	0.9	0.5–1.4

BTV, bluetongue virus; EHDV, epizootic hemorrhagic disease virus.

### BTV and EHDV RNA detection by age

**FIG. 1.**
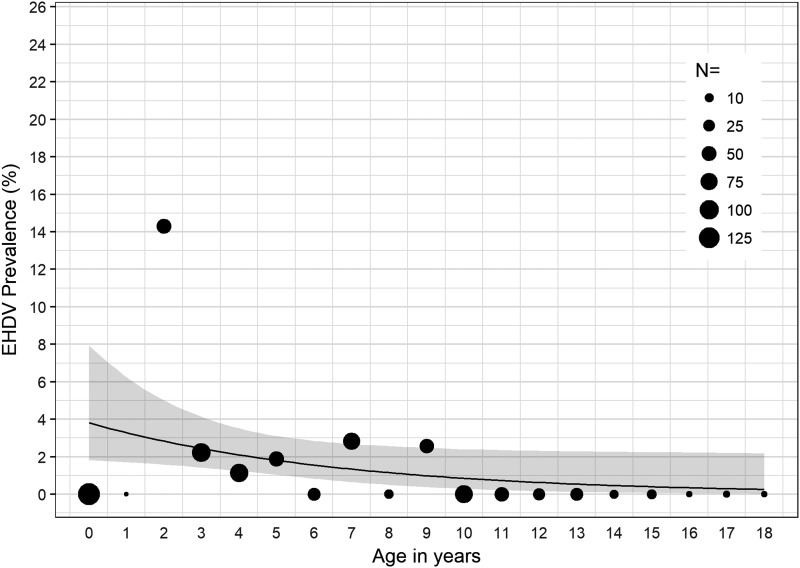
EHDV prevalence among all cattle sampled in 2016 by age in years. The estimated EHDV prevalence and 95% CI determined by the logistic regression model are represented by the *black line* and *gray band*, respectively. *Black circles*, scaled according to sample size, *N*, represent the observed EHDV prevalence within the specified age group. EHDV, epizootic hemorrhagic disease virus; CI, confidence interval.

**FIG. 2.**
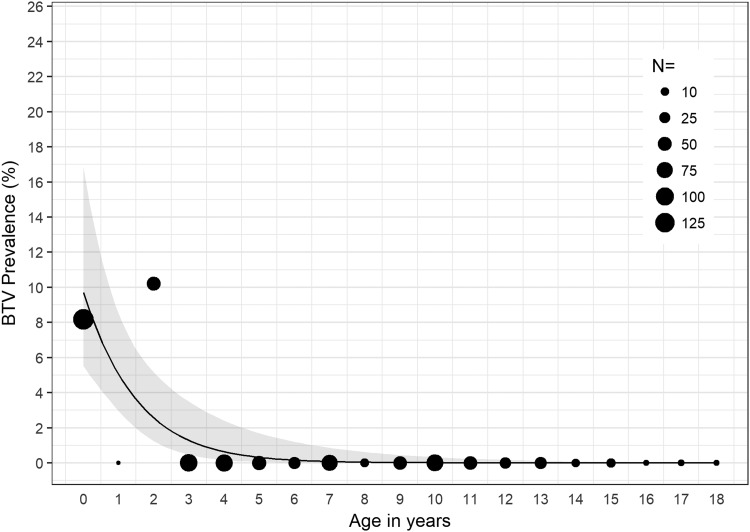
BTV prevalence among all cattle sampled in 2016 by age in years. The estimated BTV prevalence and 95% CI determined by the logistic regression model are represented by the *black line* and *gray band*, respectively. *Black circles*, scaled according to sample size, *N*, represent the observed BTV prevalence within the specified age group. BTV, bluetongue virus.

**FIG. 3.**
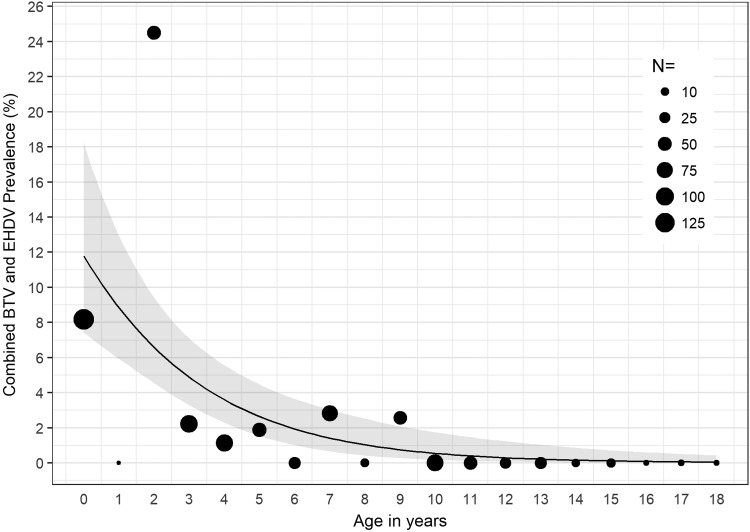
Combined BTV or EHDV prevalence among all cattle sampled in 2016 by age in years. The estimated combined BTV or EHDV prevalence and 95% CI determined by the logistic regression model are represented by the *black line* and *gray band*, respectively. *Black circles*, scaled according to sample size, *N*, represent the observed BTV or EHDV prevalence within the specified age group.

In 2016, we found significant associations between age and detection of BTV RNA (*χ*^2^ = 54.23, *p* < 0.001); age and detection of EHDV RNA (*χ*^2^ = 49.30, *p* < 0.001); and age and detection of BTV or EHDV (*χ*^2^ = 78.23, *p* < 0.001). For cattle sampled in 2016, for each 1-year increase in age, the odds of detecting EHDV in blood decreased by 14% (OR = 0.86, *p* < 0.05, 95% CI: 0.73–0.99) (Fig. 1); the odds of detecting BTV in blood decreased by 51% (OR = 0.49, *p* < 0.001, 95% CI: 0.31–0.68) (Fig. 2); and the odds of detecting either BTV or EHDV decreased by 27% (OR = 0.73, *p* < 0.001, 95% CI: 0.62–0.83) (Fig. 3).

### BTV or EHDV RNA detection by pregnancy status

We analyzed BTV and EHDV RNA detection separately and together, and found no significant association between pregnancy status and BTV, EHDV, or combined BTV or EHDV RNA detection in 2016.

## Discussion

In the United States, cattle are considered important hosts of both BTV and EHDV, although infection in cattle is largely nonclinical (Ruder et al. 2015). It has been suggested that cattle associated with research institutions be utilized for BTV and EHDV surveillance and reporting (Ruder et al. 2015). Our study highlights the potential for using cattle at research institutions to study BTV and EHDV epidemiology. In this case we partnered with a privately owned agroecological research center, on a working beef cattle ranch, to conduct surveillance. Although overall BTV and EHDV infections at our study site were low (1% and 0.9%, respectively, over all years combined), certain age groups showed high levels of infection and present opportunities for future targeted surveillance.

In 2016, >8% and >10% of calves and cattle 2 years of age, respectively, were infected with BTV, and >14% of cattle 2 years of age were infected with EHDV (Figs. 1 and 2). Detection of both BTV and EHDV was significantly associated with age, with vRNA more likely to be detected in younger than older cattle (Fig. 3). Despite the significantly higher prevalence of EHDV detection in younger versus older cattle, we did not detect EHDV from calves. However, this is likely because we did not sample calves in August or November (Supplementary Table S1), which were the months during which we detected EHDV in other age groups. Although we did not conduct serological testing in this study, it is likely the older cattle have been exposed to or previously infected with BTV and EHDV, limiting detection of vRNA in their blood due to protective immunity. Previous serological studies of cattle in Florida and Georgia have shown high prevalence of antibodies to both BTV and EHDV (Gibbs and Greiner 1985, Odiawa et al. 1985).

As noted in the methods section, sampling date and the age of cattle sampled were both opportunistic in nature. Therefore, we did not sample all ages of cattle on every date of sampling. Although an interaction effect of sampling date and age of cattle may exist, we were unable to determine that effect due to the opportunistic nature of our sampling design. Similarly, due to the unbalanced structure of the data, we may have missed detection of BTV or EHDV on some sampling dates or in some age groups, as we did not sample all age groups on all sampling dates. The ages of cattle sampled by month sampled are displayed in Supplementary Table S1.

BTV and EHDV in temperate zones have seasonal transmission patterns, usually beginning in late-summer and lasting through autumn, but transmission in the tropics may last most of the year (Maclachlan et al. 2015a, Ruder et al. 2015). Although our opportunistic study design limited our ability to analyze seasonal transmission of BTV and EHDV, we did observe differences in detection by sampling date. For example, in 2016, BTV was detected in both May and August but not in November, and EHDV was detected in August and November but not in May (Table 1). As vRNA from BTV can be detected in cattle blood months after infection, these findings may not necessarily represent contemporary transmission but instead transmission that occurred in the months prior. Our inability to sample every age group at each time point may have contributed to differences in prevalence among sampling dates (Supplementary Table S1).

Previous serological studies of cattle in Florida suggested that transmission of BTV varies from year to year (Gibbs and Greiner 1985). In our study, prevalence of BTV RNA detection was higher in 2016 compared with 2015 and 2017. We found no EHDV infection in 2015 or 2017 despite a ∼2% prevalence in 2016. However, we cannot make direct comparisons among the years as we did not sample the same range of dates each year. When looking at data from only the month of August, which was sampled 2 of the 3 years, we see lower BTV prevalence and the absence of EHDV detection in 2017 as compared with 2016.

We did not find significant associations with BTV or EHDV infection and pregnancy status. This is not surprising as clinical signs are generally not observed in BTV- or EHDV-infected cattle (Howerth et al. 2001, Savini et al. 2011, Maclachlan et al. 2015a). In addition, based on the overall low observed BTV and EHDV prevalence, the power of our study to detect these associations was very low.

## Conclusions

These results highlight the potential of domestic cattle herds as BTV and EHDV surveillance tools. The relatively high vRNA prevalence observed in cattle ≤2 years of age makes these age groups especially productive surveillance targets. Through future targeted surveillance of cattle, a more comprehensive knowledge of regional BTV and EHDV serotype and strain richness, as well seasonal and yearly patterns in BTV and EHDV transmission, may be gained. When combined with surveillance of wildlife and vector populations, surveillance of domestic cattle may provide insights into not only which serotypes and strains are present in a region but also how these may vary among sympatric ruminant populations. Knowledge gained from both virus detection and serological studies of these viruses in domestic cattle may influence best management strategies and potential vaccine targets for control in farmed and wild white-tailed deer and other domestic ruminants, which are more susceptible to disease.

## Supplementary Material

Supplemental data

## References

[B1] Ahasan MS, Subramaniam K, Lednicky JA, Loeb JC, et al. Complete genome sequence of epizootic hemorrhagic disease virus serotype 6, isolated from Florida white-tailed deer (*Odocoileus virginianus*). Genome Announc 2018; 6 10.1128/genomeA.00160-18 PMC5887027 29622607

[B2] Bonneau KR, DeMaula CD, Mullens BA, MacLachlan NJ Duration of viraemia infectious to *Culicoides sonorensis* in bluetongue virus-infected cattle and sheep. Vet Microbiol 2002; 88:115–125 12135632 10.1016/s0378-1135(02)00106-2

[B3] Brewer AW, MacLachlan NJ Ultrastructural characterization of the interaction of bluetongue virus with bovine erythrocytes in vitro. Vet Pathol 1992; 29:356–359 1325084 10.1177/030098589202900412

[B4] Canty AJ, Ripley BD Boot: Bootstrap R (S-Plus) Functions. 2016 R package version 1.3–18

[B5] Dal Pozzo F, Saegerman C, Thiry E Bovine infection with bluetongue virus with special emphasis on European serotype 8. Vet J 2009; 182:142–151 19477665 10.1016/j.tvjl.2009.05.004

[B6] Davison AC, Hinkley DV Bootstrap methods and their applications. Cambridge University Press 1997

[B7] Florida Department of Agriculture and Consumer Services. Florida's Cattle Industry. 2012

[B8] Gibbs EPJ, Greiner EC Serological observations on the epidemiology of bluetongue virus infections in the Caribbean and Florida. Prog Clin Biol Res 1985; 178:563–570 2989905

[B9] Gibbs EPJ, Greiner EC, Taylor WP, Barber TL, et al. Isolation of bluetongue virus serotype 2 from cattle in Florida: Serotype of bluetongue virus hitherto unrecognized in the Western Hemisphere. Am J Vet Res 1983; 44:2226–2228 6318609

[B10] Howerth EW, Stallknecht DE, Kirkland PD Bluetongue, epizootic hemorrhagic disease, and other orbivirus-related diseases. In: Williams ES, Barker IK, eds. Infectious Diseases of Wild Mammals 3rd ed. Ames, Iowa: Iowa State University Press, 2001:77–97

[B11] Katz J, Alstad D, Gustafson G, Evermann J Diagnostic analysis of the prolonged bluetongue virus RNA presence found in the blood of naturally infected cattle and experimentally infected sheep. J Vet Diagn Investig 1994; 6:139–142 8068742 10.1177/104063879400600201

[B12] Kedmi M, Van Straten M, Ezra E, Galon N, et al. Assessment of the productivity effects associated with epizootic hemorrhagic disease in dairy herds. J Dairy Sci 2010; 93:2486–2495 20494156 10.3168/jds.2009-2850

[B13] Maclachlan NJ, Mayo CE, Daniels PW, Savini G, et al. Bluetongue. Rev Sci Tech 2015a; 34:329–340 26601438 10.20506/rst.34.2.2360

[B14] MacLachlan NJ, Nunamaker RA, Katz JB, Sawyer MM, et al. Detection of bluetongue virus in the blood of inoculated calves: Comparison of virus isolation, PCR assay, and in vitro feeding of *Culicoides variipennis*. Arch Virol 1994; 136:1–8 8002778 10.1007/BF01538812

[B15] Maclachlan NJ, Zientara S, Savini G, Daniels PW Epizootic haemorrhagic disease. Rev Sci Tech 2015b; 34:341–351 26601439 10.20506/rst.34.2.2361

[B16] Mellor PS, Boorman J, Baylis M Culicoides biting midges: Their role as arbovirus vectors. Annu Rev Entomol 2000; 45:307–340 10761580 10.1146/annurev.ento.45.1.307

[B17] Odiawa G, Blue JL, Tyler DE, Shotts EB Bluetongue and epizootic hemorrhagic disease in ruminants in Georgia: Survey by serotest and virologic isolation. Am J Vet Res 1985; 46:2193–2196 2998240

[B18] R Core Team. R: A language and environment for statistical computing. R Foundation for Statistical Computing Vienna, Austria 2016 http://www.R-project.org/

[B19] Ruder MG, Lysyk TJ, Stallknecht DE, Foil LD, et al. Transmission and epidemiology of bluetongue and epizootic hemorrhagic disease in North America: Current perspectives, research gaps, and future directions. Vector-Borne Zoonotic Dis 2015; 15:348–363 26086556 10.1089/vbz.2014.1703

[B20] Savini G, Afonso A, Mellor P, Aradaib I, et al. Epizootic haemorragic disease. Res Vet Sci 2011 p. 1–17 10.1016/j.rvsc.2011.05.004 21665237

[B21] Schirtzinger EE, Jasperson DC, Ostlund EN, Johnson DJ, et al. Recent US bluetongue virus serotype 3 isolates found outside of Florida indicate evidence of reassortment with co-circulating endemic serotypes. J Gen Virol 2018; 99:157–168 29120297 10.1099/jgv.0.000965 PMC5882081

[B22] Schwartz-Cornil I, Mertens PPC, Contreras V, Hemati B, et al. Bluetongue virus: Virology, pathogenesis and immunity. Vet Res 2008; 39:46 18495078 10.1051/vetres:2008023

[B23] Subramaniam K, Lednicky JA, Loeb J, Sayler KA, et al. Genomic sequences of epizootic hemorrhagic disease viruses isolated from florida white-tailed deer. Genome Announc 2017; 5 10.1128/genomeA.01174-17 PMC5658499 29074661

[B24] Swain HM, Boughton EH, Bohlen PJ, Lollis LOG Trade-offs among ecosystem services and disservices on a Florida ranch. Rangelands 2013; 35:75–87

[B25] Wernike K, Hoffmann B, Beer M Simultaneous detection of five notifiable viral diseases of cattle by single-tube multiplex real-time RT-PCR. J Virol Methods 2015; 217:28–35 25746154 10.1016/j.jviromet.2015.02.023

