# Supplementary material for: Epidemiology of Bluetongue Virus and Epizootic Hemorrhagic Disease Virus in Beef Cattle on a Ranch in South-Central Florida
Source: Vector Borne Zoonotic Dis. 2019 Sep 26;19(10):752–7. doi: 10.1089/vbz.2018.2406 (PMC6765206; doi:10.1089/vbz.2018.2406)
Supplement: Supplemental data [file Supp_Table1.pdf]

Supplementary Data

Supplementary Table S1. Number of Cattle Sampled by Age and Month in 2016

	Month sampled								
	May			August			November		
Age in years	No. sampled	No. BTV positive (%)	No. EHDV positive (%)	No. sampled	No. BTV positive (%)	No. EHDV positive (%)	No. sampled	No. BTV positive (%)	No. EHDV positive (%)
<1	110	9 (8)	0 (0)	0	—	—	0	—	—
1	0	—	—	1	0 (0)	0 (0)	0	—	—
2	1	0 (0)	0 (0)	46	5 (11)	7 (15)	2	0 (0)	0 (0)
3	0	—	—	17	0 (0)	0 (0)	73	0 (0)	2 (3)
4	1	0 (0)	0 (0)	36	0 (0)	1	50	0 (0)	0 (0)
5	4	0 (0)	0 (0)	45	0 (0)	1	4	0 (0)	0 (0)
6	4	0 (0)	0 (0)	9	0 (0)	0 (0)	16	0 (0)	0 (0)
7	15	0 (0)	0 (0)	25	0 (0)	1	31	0 (0)	1 (3)
8	5	0 (0)	0 (0)	1	0 (0)	0 (0)	5	0 (0)	0 (0)
9	9	0 (0)	0 (0)	6	0 (0)	0 (0)	24	0 (0)	1 (4)
10	23	0 (0)	0 (0)	13	0 (0)	0 (0)	44	0 (0)	0 (0)
11	11	0 (0)	0 (0)	6	0 (0)	0 (0)	30	0 (0)	0 (0)
12	13	0 (0)	0 (0)	8	0 (0)	0 (0)	6	0 (0)	0 (0)
13	10	0 (0)	0 (0)	13	0 (0)	0 (0)	8	0 (0)	0 (0)
14	4	0 (0)	0 (0)	3	0 (0)	0 (0)	3	0 (0)	0 (0)
15	6	0 (0)	0 (0)	5	0 (0)	0 (0)	1	0 (0)	0 (0)
16	2	0 (0)	0 (0)	0	—	—	0	—	—
17	1	0 (0)	0 (0)	3	0 (0)	0 (0)	0	—	—
18	2	0 (0)	0 (0)	0	—	—	0	—	—
NA[a]	17	0 (0)	0 (0)	5	0 (0)	1	15	—	—
All ages	238	9 (4)	0 (0)	242	5 (2)	11 (5)	312	0 (0)	4 (1)

[a]Animals for which age was not recorded.
BTV, bluetongue virus; EHDV, epizootic hemorrhagic disease virus.
